# HARNESSING THE NETWORK OF GERONTOLOGY IN HIGHER EDUCATION TO IMPROVE RECRUITMENT STRATEGIES

**DOI:** 10.1093/geroni/igz038.2283

**Published:** 2019-11-08

**Authors:** Jacqueline Eaton, Margaret B Neal

**Affiliations:** 1 University of Utah, Salt Lake City, Utah, United States; 2 Portland State University, Portland, Oregon, United States

## Abstract

While the workforce requires applicants trained to meet the needs of an aging population, many gerontology programs find recruiting students a challenge. Barriers to enrollment vary and are influenced by disciplinary silos, miscomprehension of terminology (such as “gerontology”), vague career paths, and limited resources. Program directors are challenged to identify innovative and new strategies that market training opportunities at a variety of levels and across colleges and departments. In an effort to harness the power of networks, it is our goal that this symposium fosters discussion in order to improve our communal approach to increase the number of individuals prepared for careers in aging. This symposium examines the recruitment experiences and challenges from the perspective of gerontology programs at four universities. Fruhauf (Colorado State University) explores the importance of university-community engagement in support of gerontology programs. Fauth and Liu (Utah State University) describe effective recruitment strategies for an interdisciplinary gerontology certificate housed within a department of Human Development and Family Studies. Eaton (University of Utah) presents the outcomes from a targeted initiative to increase early undergraduate enrollment in an interdisciplinary online gerontology minor. Finally, Yorgason (Brigham Young University) shares a systematic approach to leverage resources and fundraising that successfully increased program enrollment and faculty involvement throughout campus. Dr. Margaret B. Neal, an experienced educator and former director of the Institute on Aging at Portland State University, will facilitate discussion surrounding how we can mobilize our networks to strengthen, innovate, and expand approaches to recruitment in gerontology.

